# Maintaining disorder: estimating the association between policing and psychiatric hospitalization among youth in New York City by neighborhood racial composition, 2006–2014

**DOI:** 10.1007/s00127-024-02738-7

**Published:** 2024-08-01

**Authors:** Samuel E. Packard, Zoe Verzani, Megan C. Finsaas, Natalie S. Levy, Ruth Shefner, Arrianna M. Planey, Amelia K. Boehme, Seth J. Prins

**Affiliations:** 1 Department of Epidemiology, Columbia University Mailman School of Public Health, New York, NY USA; 2 Department of Sociomedical Sciences, Columbia University Mailman School of Public Health, New York, NY USA; 3 Department of Health Policy and Management, Gillings School of Global Public Health, University of North Carolina, Chapel Hill, NC USA; 4 Department of Neurology, Columbia University, New York, NY USA

**Keywords:** Mental Health, Police, Race/Ethnicity, Adolescent Psychiatry, Social determinants of Health

## Abstract

**Purpose:**

To assess whether neighborhood-level measures of policing are spatio-temporally associated with psychiatric hospialization among adolescents and young adults in New York City, and whether this association varies by neighborhood racial composition.

**Methods:**

We derived population-based measures of policing from the New York City Police Department (NYPD), psychiatric hospitalization from Statewide Planning and Research Cooperative System (SPARCS) data, and socio-demographic data from the American Community Survey (ACS), aggregated by month and ZIP Code Tabulation Area (ZCTA) from 2006 to 2014. Multi-level negative binomial regression models assessed hospitalization-time of youth aged 10–24 as the dependent variable and the rate of policing events as the primary independent variable, adjusting for neighborhood poverty, unemployment, and educational attainment. Multiplicative interaction was assessed between policing and tertiles of the percentage of Black residents.

**Results:**

A total of 11,900,192 policing incidents and 2,118,481 person-days of hospitalization were aggregated to 19,440 ZCTA-months. After adjusting for neighborhood-level sociodemographic characteristics, an increase in one policing incident per 1,000 residents was associated with a 0.3% increase in the rate of youth psychiatric hospitalization time (IRR 1.003 [1.001–1.005]). Neighborhood racial composition modified this effect; not only was the rate of psychiatric hospitalization and policing higher in neighborhoods with a higher proportion of Black residents, but the association between these was also significantly higher in neighorhoods with a larger share of Black residents compared with predominantly non-Black neighborhoods.

**Conclusion:**

Neighborhoods experiencing higher rates of policing during the study period experienced higher burdens of psychiatric hospitalization among adolescent and young adult residents. This association was larger in neighborhoods of color which have been disproportionately targeted by “hot spot” and order-maintenance policing practices and policies.

**Supplementary Information:**

The online version contains supplementary material available at 10.1007/s00127-024-02738-7.

## Introduction

People who live, work, and spend time in aggressively policed neighborhoods are regularly exposed to police through either direct encounters or witnessing encounters involving friends, family, and neighbors. These traumatic and often violent events are frequently experienced by adolescents, who are in a critical developmental period associated with substance use initiation and psychiatric symptom onset [1–5]. In New York City (NYC), at least half of all recorded police stops involve individuals under age 25, and among at-risk youth nationwide who have been involved in a police stop, 40% report their first encounter at age 13 or younger [6, 7]. Not only is exposure to policing prevalent among adolescents, but it varies by racial group membership. In the United States, roughly 23-29% of adolescents aged 12–18 have been stopped by police at least once, and roughly 21% of Black boys, compared with 8.6% of white boys, have been stopped two or more times [8]. Building on a growing body of evidence finding that exposure to aggressive policing is a social determinant of adolescent mental health [8–12], the present study seeks to assess whether policing is spatio-temporally associated with a severe adolescent mental health outcome, psychiatric hospitalization.

There is mounting empirical evidence that both direct [6, 8, 13–23] and indirect (e.g., vicarious) [19, 21, 24] exposure to policing is associated with individual- and community-level adolescent mental health, and that this phenomenon is racialized [25]. For example, at the individual level, police stops, have been linked to depressive symptoms among adolescents, and this association is twice as strong in young Black men as compared with young white men [26]. A mediation analysis by Boen and colleagues found that higher rates of police contact among young men of color account for disparities in depressive symptoms between them and their white counterparts [13]. A systematic review of studies focused specifically on the health of young Black men found that police contact is consistently linked to stress and other self-reported outcomes such as anger, sadness, and psychological distress [27].

Regarding indirect or vicarious exposure to policing, witnessing police encounters is associated with feelings of anger and insecurity, elevated emotional and psychological distress, and suicidality. [6, 21, 24] These associations are also racialized: witnessing police stops secondhand is more strongly associated with emotional distress and lower self-reported health among youth of color than in non-Hispanic white youth [24, 28]. At the community level, exposure to policing varies significantly across places where people live.

Over-policing is observed in communities which are more likely to face high levels of material and social deprivation, [29, 30] which are, in turn, associated with incidence and prevalence of serious mental illness and psychiatric hospitalizations in adolescents and adults. [31–36]. Most medium to large police agencies adopt one or more strategies of “order maintenance” policing, in which police resources are pre-emptively focused on certain areas, based on purported and contested theories of deterrence [10]. These areas are generally where poor and working class communities of color reside.New York City was an early and proactive adopter of “order maintenance” approaches to policing. Stop, question, and frisk (SQF) practices were expanded in the mid-1990s. Since the program first started producing these data, this “aggressive approach to low level disorder” was demonstrated to disproportionately target people of color [37]. Prior to SQF practices being declared unconstitutional in 2013, the New York City Police Department was conducting a half million such stops per year. Across New York City, 29% of adults report ever being stopped, searched, or questioned by police. While the lifetime prevalence of ever being stopped or questioned by police is comparable between white and Black adults, rates of physical threats and abuse during these encounters is three times higher among Black adults compared with their white counterparts, and 80% of stops recorded under SQF involved Black or Hispanic people [38, 39]. At the city level, this targeting of minoritized communities as surveillance “hot spots” results in spatially and racially patterned police activity across neighborhoods. An analysis by Lautenschlager and Omori which examined the incidence and severity of stops involving use of force in NYC found both to be concentrated in Black census tracts, a pattern which was stable over a ten-year period and independent of disadvantage, residential instability, racial/ethnic heterogeneity, and actual and perceived crime [40]. They concluded that patterns of policing amount not just to the aggregate of encounters between individuals and police, but characteristics of neighborhoods resulting from multiple social processes, empirically corroborating Gilmore’s description of concentrated police surveillance as a “prime organizing principle of everyday life” in those communities which are the focus of these order-maintenance and “hot-spot” policing practices [41, 42].

Population-level rates of severe mental health events such as emergency room visits, hospitalizations, and suicides are also spatially patterned [43, 44]. Like encounters with police, neighborhood-level spatial patterns in psychiatric outcomes are important to study from a public health perspective. While each severe mental health event is itself a profound and potentially destabilizing life event for the people involved, the clustering of mental health emergencies over time and space reflects the social and political arrangements that produce them. Indeed, people’s physical and social environments are determinants of mental health outcomes [44, 45]. Measures of socio-economic status as well as indices of socio-economic deprivation and social fragmentation are associated with neighborhood-level rates of psychiatric inpatient admissions in large urban centers, including New York City [46, 47]. However, these broad measures may not fully characterize aspects of place that are consequential for mental health [48, 49]. Evidence that exposure to the criminal legal system is harmful for adolescent mental health suggests that the spatial patterning of policing may also be an important driver of the differential burden of mental illness between neighborhoods within a city.

Given that (1) direct and indirect exposure to policing may be a stressful and traumatic experience, (2) such experiences are known triggers of serious mental illness onset, [50, 51], and (3) higher levels of direct and indirect exposure to policing takes place in communities already at higher risk of serious mental illness and psychiatric hospitalization, it follows that higher community levels of policing may predict higher rates of psychiatric hospitalization. In this study, we operationalized the geospatial density of policing as a contextual aspect of the social environment that operates as a distinct and racialized structural determinant of severe mental health outcomes among youth, independent of area measures of socio-economic status. We examined the spatial association between neighborhood-level rates of policing and psychiatric hospitalizations among adolescents and young adults across New York City using longitudinal data from 2006 to 2014, and further explored whether this association differed across neighborhoods according to racial composition, accounting for neighborhood-level socio-economic characteristics. Specifically, we hypothesized that neighborhood-level rates of policing are positively associated with rates of psychiatric hospitalization time among adolescents and young adults after controlling for measures of socio-economic status, and that neighborhood racial composition modifies the effect of this association.

## Methods

### Study design and population

We used data on inpatient hospital admissions among New York City residents aged 10–24, records of policing incidents involving New York City residents of all ages, and American Community Survey (ACS) data representing neighborhood sociodemographic characteristics. The time period 2006–2014 was selected for common data availability between the linked datasets and because it covered the bulk of the period during which SQF was in full effect.

### Data and measures

#### Exposure variables: policing encounters

We used publicly available data from the New York City Open Data portal. Separate datasets provided by the New York City Police Department (NYPD) contained records of all documented (1) SQF encounters, (2) arrests, and (3) criminal court summonses, with common data elements such as the age group and reported race and ethnicity of the person involved as well as the geolocation (latitude/longitude) where the incident occurred [52–54]. We calculated the rate of policing incidents as the total number of combined policing events per 1,000 people per month for each Zip Code Tabulation Area (ZCTA). We also accessed data for complaints made to the NYPD for reported crimes and criminal behavior, similarly calculating the rate per 1,000 people per month for each ZCTA [55].

#### Outcome variable: psychiatric hospitalization

We obtained data on inpatient hospital admissions and hospital location from the New York State Department of Health Statewide Planning and Research Cooperative System (SPARCS), an all-payer reporting system providing patient-level data on hospital discharges [56]. Hospitalizations were classified as psychiatric if the record contained an International Classification of Disease, Version 9 (ICD-9) code 290–316 as either a primary or contributing diagnoses, consistent with a definition used by the New York City Department of Health and Mental Hygiene [57].

Length of stay was calculated for each hospitalization as the difference between the admission and discharge dates, expressed as inpatient days. We calculated the rate of psychiatric hospital inpatient days per 1,000 residents age 10–24 per month for each ZCTA, based on patient home of record. While previous studies have utilized both person-time of hospitalization and discrete counts of hospital inpatient admissions as outcome measures, we chose inpatient days as the primary outcome measure for the present study. The average length of stay for psychiatric hospitalizations among youth in New York City is three times that of non-psychiatric hospitalizations, resulting in significant variation in the length of stay for mental health related admissions and suggesting that the amount of time hospitalized is a significant aspect of these encounters, potentially serving as a proxy for severity [57]. In this study, although we were concerned with the racialized impact of policing on adolescent mental health, race/ethnicity variables we did not calculate rates of psychiatric hospitalizations separately by individual patient race/ethnicity as these variables in the SPARCS data have been previously reported as unreliable and not suitable for imputation in studies of racially diverse neighborhoods [57, 58]. Indeed, one third of the hospital admissions in our dataset had missing values for race. Thus, we did not calculate rates of psychiatric hospitalization by race/ethnicity at the individual level of the patient, but rather by neighborhood composition as described below.

#### Neighborhood demographic and socio-economic characteristics

Neighborhood socio-economc and demographic data including total population, proportions of residents in each age group and self-reported race category, percentage of households below the poverty line, and rates of adult educational attainment and unemployment rate were obtained at the ZCTA level from the American Community Survey (ACS) 5-year estimates. Additional variables were accessed to construct indices of socioeconomic deprivation and social fragmentation, described in detail in the supplementary material. Average distance to inpatient hospital services, a predictor of healthcare service utilization in prior studies, was estimated as the distance between the ZCTA geographic centroid and the nearest hospital.

We defined residents of color as those who were categorized as any race and ethnicity other than non-Hispanic white and Black residents as those categorized as non-Hispanic Black. The proportion of the total population represented by each of these categories was calculated for each ZCTA, and categorized in tertiles. Racial neighborhood composition in this study serves as a proxy for the racialization of neighborhoods and the people living within them, representing multiple processes of oppression, exploitation, and inequality, such as historical policies of racial residential segregation and the well-documented overpolicing of minoritized people and neighborhoods [38, 59–62].

To account for demographic changes over the study and to provide time-varying covariate and population denominator data for the longitudinal models, we obtained ACS 5-year estimates for 3 different periods, which we matched to exposure and outcome data (years 2006–2008 were matched to the ACS 2007–2011 file, years 2009–2011 were matched to the ACS 2009–2013 file, and years 2012–2014 were matched to the ACS 2011–2015 file). ACS 5-year estimates were used because 1-year estimates were not available for ZCTA geography. For example, neighborhood socio-demographic data for the year-month observation corresponding to January 2010 was taken from ACS 2009–2013 5-year estimates. We accessed American Community Survey Data and ZCTA boundaries with the tidycensus and tigris packages for R [63, 64].

### Statistical analysis

#### Modeling strategy

The resulting data represented monthly time-varying measures of policing and hospitalizations, as well as triennial time-varying socio-economic and demographic characteristics, for 180 residential ZCTAs over the 108 months between 2006 and 2014. To model the association between repeated measures of policing and hospitalization person-time at the ZCTA level while accommodating for different baseline rates and clustering within neighborhoods, we fit multi-level negative binomial models regressing monthly inpatient days among 10- to 24-year-olds on the rate of policing incidents. We included the natural log of the proportion of residents aged 10–24 as the offset term and specified random intercepts and slopes for ZCTA varying by month. We adjusted for the confounders described above: household poverty rate, percentage unemployed residents over age 16, and percentage of adults 25 and older with less than high school educational attainment. We also tested whether neighborhood racial composition—as a proxy for racialized social inequality—modified the association between policing and adolescent psychiatric hospitalizations on the multiplicative scale by including an interaction term representing the cross-product of tertiles of the proportion of Black residents and the policing rate.

#### Sensitivity analyses

We performed several sensitivity analyses by replicating the models described above under the following conditions: first, the outcome definition was restricted to include only inpatient admissions with a psychiatric ICD-9 code as the primary diagnosis, excluding those records in which a psychiatric condition only appeared as a contributing diagnosis. Second, a more comprehensive index of social fragmentation, described in the supplementary material, was used as a covariate in place of separate continuous measures of poverty, education, and unemployment. Third, the exposure was restricted to only pedestrian stops and criminal summonses to better approximate “order maintenance” or “broken windows” policing [65]. Fourth, the rate of hospitalizations based on counts of inpatient admissions was used rather than the rate of total hospitalization time. Fifth, hospitalization records with a length of stay greater than one month were excluded to reduce the influence of outliers. Sixth, the exposure was lagged such that policing predicts future hospitalization, at 1 month and 3 months post-exposure. Lastly, we modeled the relationship between policing and hospitalization controlling for the rate of crime complaints as a covariate.

## Results

### Distribution of exposure, outcome, and covariate data

Table 1 summarizes the neighborhood characteristics of the 180 residential ZCTAs included in the analysis by the three periods of American Community Survey data. While there was a citywide increase in total population during the study period, there was an overall decrease in the proportion of adolescent and young adult residents. Table 2 summarizes exposure, outcome, and covariate measures by neighborhood racial composition. These summary statistics show that neighborhood levels of the exposure, outcome, and covariates are positively associated with the proportion of Black residents at the neighborhood level.

**Table 1:** Selected demographic characteristics of New York City neighborhoods, 2006–2014. Estimates derived from American Community Survey 5-year estimates for the three time periods used in the study

	ACS 5-year estimate period Mean (SD)		
Characteristic	2007–2011	2009–2013	2012–2016
Total Population	45,310 (26,014)	46,089 (26,452)	47,161 (27,055)
% Age 10–17	9.1 (3.4)	8.8 (3.2)	8.4 (3.0)
% Age 18–24	10.1 (3.0)	10.0 (3.0)	9.3 (2.9)
% Household Poverty	16.9 (10.3)	17.7 (10.4)	17.7 (10.2)
Median Household Income	62,520 (31,616)	63,864 (32,223)	67,842 (34,398)
% Education < High School	18.7 (11.2)	18.1 (11.0)	17.1 (10.5)
% Adults Unemployed	9.2 (3.5)	10.3 (3.8)	8.2 (3.4)
% Residents of Color	64.6 (28.5)	65.4 (28.4)	66.5 (27.4)
% Black Residents	22.6 (26.2)	22.3 (25.8)	21.7 (25.2)
Distance to Hospital (Miles)	0.6 (0.7)	0.6 (0.7)	0.6 (0.7)
Rate of Psychiatric Hospitalizations (per 1,000 residents age 10–24 per year)	13.1 (9.0)	14.3 (7.4)	15.3 (6.7)
Rate of Psychiatric Inpatient Days (per 1,000 residents age 10–24 per year)	110.0 (97.9)	113.0 (97.9)	106.4 (87.4)
Rate of Policing Incidents (per 1,000 residents of all ages per year)	174.1 (175.4)	186.1 (162.7)	126.4 (111.2)

**Table 2:** Demographic, psychiatric hospitalization, and policing measures of New York City neighborhoods by tertiles of neighborhood racial composition, 2006–2014. Estimates derived from American Community Survey 5-year estimates

Characteristic	ZCTA % Black Residents Mean (SD)		
	Tertile 1 0–4%	Tertile 2 4–25%	Tertile 3 25–93%
% Age 10–17	7.0 (3.1)	7.8 (2.9)	11.2 (1.9)
% Age 18–24	8.0 (3.1)	10.3 (3.0)	11.1 (1.6)
% Household Poverty	10.6 (6.2)	16.4 (7.3)	24.5 (10.8)
% Education < High School	11.7 (9.7)	17.5 (9.9)	23.4 (9.2)
% Adults Unemployed	6.7 (1.6)	8.7 (2.8)	12.1 (3.1)
% Black Residents	2.3 (1.1)	11.3 (6.5)	51.9 (21.7)
Distance to Hospital (Miles)	0.6 (0.7)	0.5 (0.7)	0.6 (0.8)
Rate of Psychiatric Hospitalizations (per 1,000 residents age 10–24 per year)	14.2 (10.8)	14.0 (7.4)	15.9 (4.7)
Rate of Psychiatric Inpatient Days (per 1,000 residents age 10–24 per year)	60.5 (53.1)	88.3 (60.6)	174.2 (108.8)
Rate of Policing Incidents (per 1,000 residents of all ages per year)	90.8 (82.1)	172.6 (171.9)	220.4 (128.3)

**Fig. 1:**
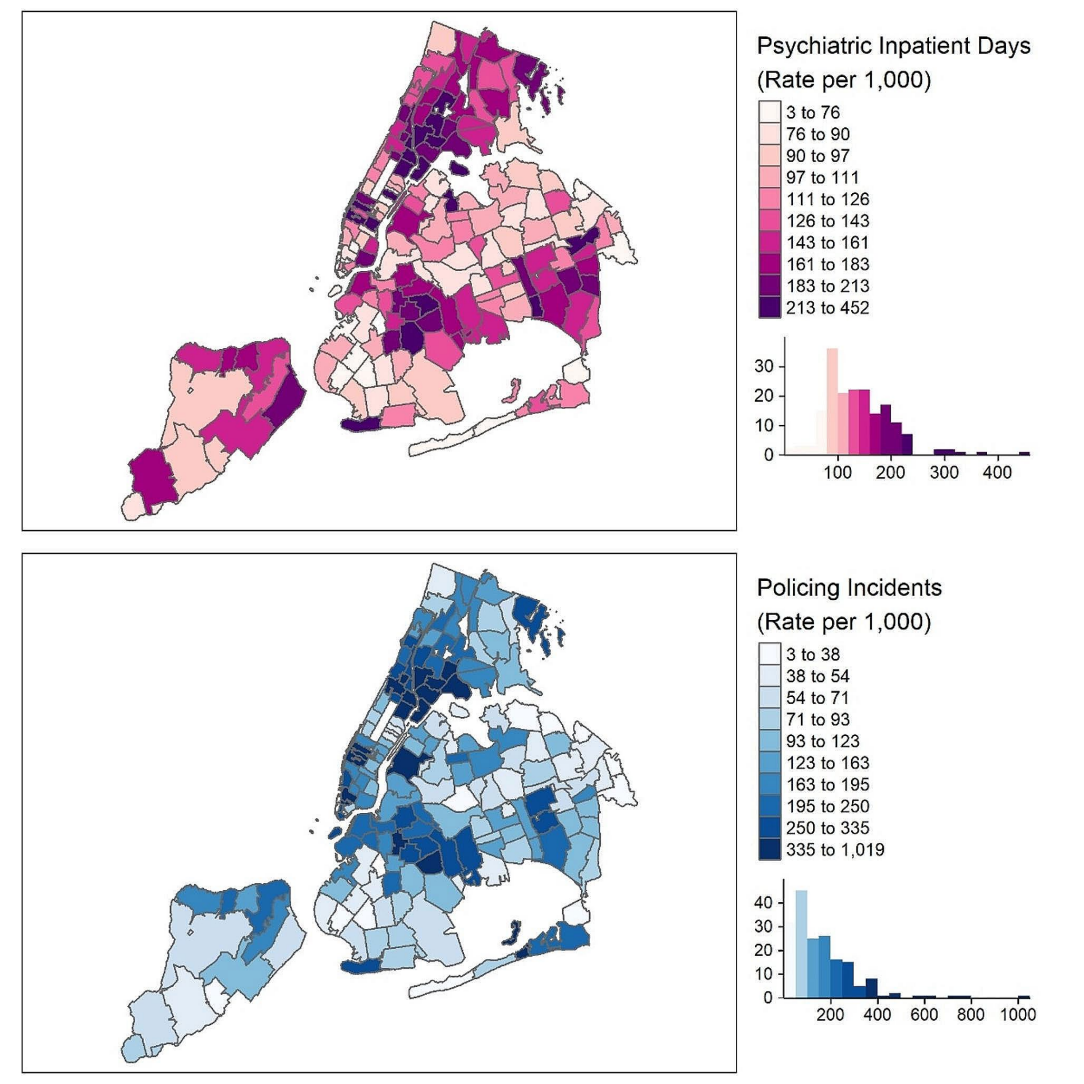
Geospatial distribution of psychiatric inpatient days (top) and policing incidents (bottom) in New York City from 2006–2014. Rates are calculated as the average number of inpatient days per 1,000 residents age 10–24 per year and policing incidents per 1,000 residents of all ages per year, based on ACS 5-Year Population Estimates for 2009–2013

During this time period, there were 11,900,192 total recorded policing incidents, including 3,577,583 arrests, 4,308,026 criminal summonses, and 4,014,583 stops under the SQF program. There were 194,834 psychiatric inpatient admissions observed among adolescents and young adults aged 10–24, representing 2,118,481 person-days of hospitalization. We aggregated these policing incidents and psychiatric hospitalizations to the 180 included residential ZCTAs for each month in the study period, resulting in 19,440 ZCTA-months from 2006 to 2014. Figure 1 shows geospatial and temporal distributions of the exposure and outcome measures, demonstrating substantial between-neighborhood variation and a high degree of correlation between rates of policing and rates of psychiatric hospitalization.

The most common primary diagnoses among the psychiatric hospitalizations are summarized in Table S-1. Most (56%) hospitalizations had a psychiatric ICD-9 code listed as the primary diagnosis, with episodic mood disorders as the most frequent diagnoses, a category that includes bipolar and major depressive disorders. Among those hospitalizations with a psychiatric code listed as a contributing diagnosis, the most common primary diagnoses were epilepsy (ICD-9 345, 3.5%), general symptoms (ICD-9 780, 1.8%), and asthma (ICD-9 493, 1.8%).

### Primary analysis

**Fig. 2:**
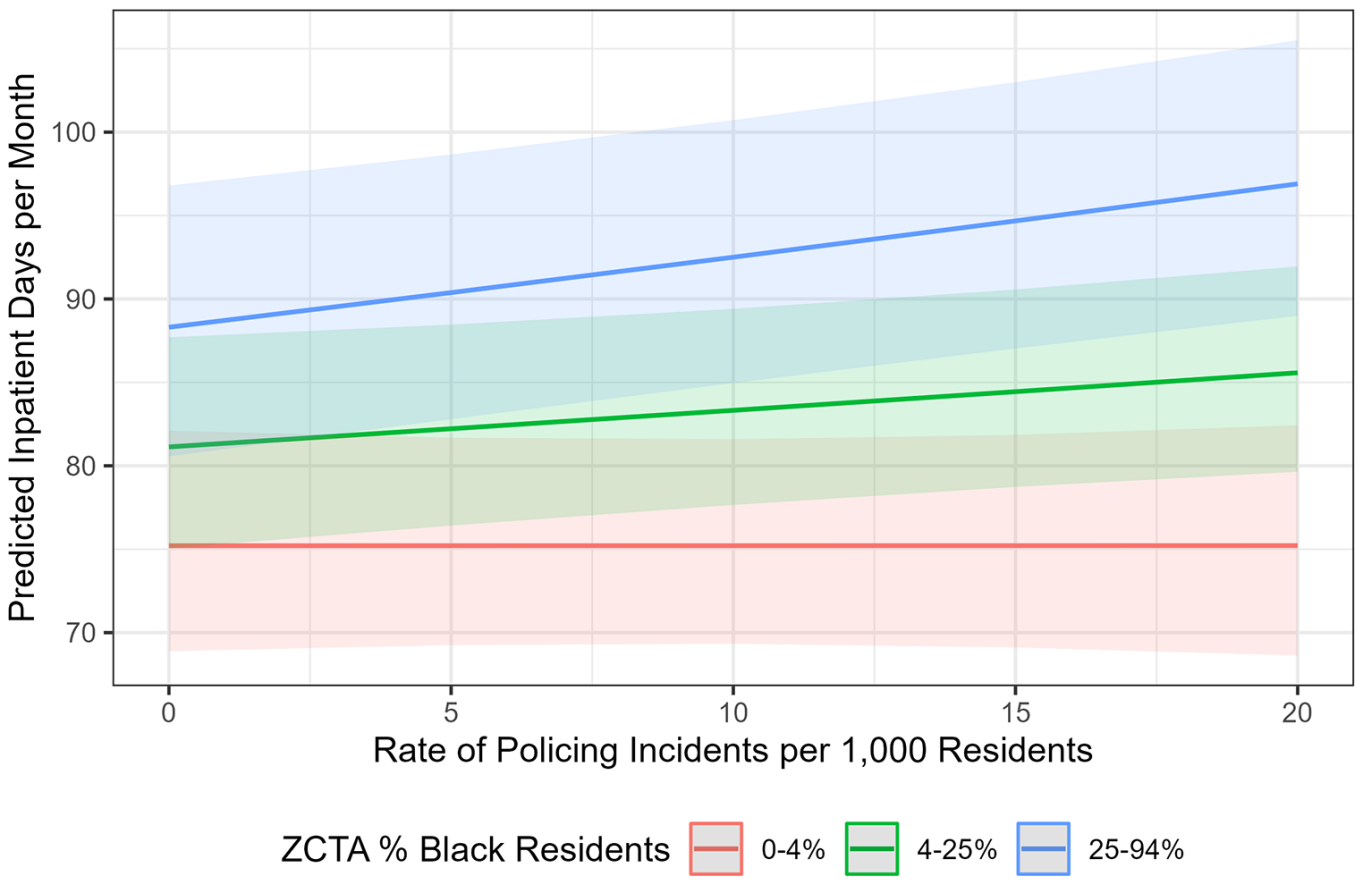
Association of the rate of policing incidents and the estimated total burden of psychiatric inpatient days among 10–24 year olds per month by neighborhood racial composition. Rate of policing calculated as incidents per 1,000 residents per month

Table 3 shows that after adjusting for neighborhood poverty, education, and unemployment, an increase in one policing incident per 1,000 residents was associated with a 0.3% increase in the rate of youth psychiatric hospitalization time (IRR 1.003 [1.001–1.005]). For instance, in two socio-economically comparable neighborhoods with 7,500 youth aged 10–24 who experienced a total of 75 psychiatric inpatient days in a given month (10 inpatient days per 1,000 youth), a difference in 10 policing incidents per 1,000 residents would be associated with a difference in 2.5 inpatient days each month, or 30 per year. Neighborhood racial composition modified this effect; not only did neighborhoods with a higher proportion of Black residents have more psychiatric hospitalization days on average, but the association of this outcome with policing was significantly different in these neighborhoods compared to predominantly non-Black neighborhoods. The difference in average predicted values for hospitalization time associated with policing by neighborhood racial composition is visualized in Fig. 2.

**Table 3:** Estimates from fully-adjusted multilevel models demonstrating the association between policing rates and the rate of psychiatric hospitalization time, and effect modification by neighborhood racial composition

	Model 1		Model 2	
*Predictors*	*IRR*	*CI*	*IRR*	*CI*
Policing Rate	1.003	1.001–1.005	1.000	0.996–1.004
Black Residents (1st Tertile)			*ref*	*ref*
Black Residents (2nd Tertile)			1.079	0.980–1.188
Black Residents (3rd Tertile)			1.174	1.033–1.334
Policing Rate X Black Residents (1st Tertile)			*ref*	*ref*
Policing Rate X Black Residents (2nd Tertile)			1.003	0.998–1.007
Policing Rate X Black Residents (3rd Tertile)			1.005	1.000–1.009

Model 1 includes only a fixed effect of the policing rate, while Model 2 includes an interaction term between this rate and tertiles of neighborhood racial composition. Both models are adjusted for neighborhood poverty, education, and unemployment rate. The coefficient represents an increase in the rate of inpatient days among 10–24 year olds associated with a one-unit increase in the rate of policing incidents per 1,000 people per month in the general population. *IRR = Incidence Rate Ratio*,* CI = 95% Confidence Interval*

#### Sensitivity analyses

Results from the eight sensitivity analyses do not appreciably alter the findings of the primary analysis and are summarized in Tables S2 - S9. Substituting composite indices of socio-economic deprivation and social fragmentation for separate continuous measures of education, poverty, and unemployment produced similar estimates. Restricting the outcome to hospitalizations with a psychiatric primary diagnosis and restricting the exposure to only pedestrian stops and criminal summonses resulted in substantially stronger interaction coefficients for the racialized association between policing and psychiatric hospitalization time. The remaining sensitivity analyses resulted in slightly attenuated or less precise estimates, including modeling the count of inpatient admissions rather than hospitalization days, removing outliers for unusually long hospitalizations, modeling the lagged association between policing at t0 and hospitalization time at t1 or t3, and controlling for public complaints of criminal activity as a covariate. While these sensitivity analyses had heterogenous results, they collectively indicate that the observed association between exposure to policing and the serious psychiatric outcomes is not explained away by potential confounding factors, nor an artifact of a particular operationalization or extreme cases in our data.

## Discussion

In this study, we linked longitudinal data from multiple sources to assess whether the geospatial density of policing was positively associated with the amount of time that adolescents and young adults spent hospitalized for psychiatric emergencies across New York City neighborhoods from 2006 to 2014. Our results demonstrate that neighborhoods experiencing higher rates of policing during the study period also experienced higher burdens of psychiatric hospitalization among their adolescent and young adult residents, an association independent of neighborhood socio-economic characteristics. We further demonstrated that this effect is modified by neighborhood racial composition: the magnitude of the association between policing and adolescent psychiatric hospitalizations is larger in neighborhoods of color compared with predominantly white neighborhoods.

These results build on two bodies of research in social and psychiatric epidemiology. First, we add to the growing body of evidence on the role of systemic racism and the carceral state as social determinants of mental health, and racialized disparities therein. Building on studies showing that individual-level exposure to police increases risk for negative mental health outcomes, we find that community-level rates of policing are also associated with higher rates of severe psychiatric outcomes. While this finding may be due to the collective impact of direct or vicarious exposure to policing on the psychiatric outcomes of individual adolescents, it may also be due in part to contextual effects that policing has on neighborhoods. In other words, racial disparities in the total burden of time that adolescents and young adults spend hospitalized for psychiatric care may reflect, among other things, the disproportionate targeting of communities of color by police. This is consistent with the notion that while aggressive policing tactics can result in traumatic experiences for individual people, they also constitute a broader form of social harm as a contextual aspect of the neighborhood environment. This has been articulated in qualitative research on the subject, such as Wendel and colleague’s study of Black youth’s perceptions of police violence in West Louisville, Kentucky, in which the physical and psychological violence of policing experienced by Black youth is interpreted as a form of community-level structural violence, defined as the “social arrangements that put individuals and communities in harm’s way” [66, 67].

Second, these findings contribute to the literature on social and structural determinants of neighborhood-level variation in severe psychiatric outcomes among adolescents and young adults. Building on prior evidence that simply witnessing an arrest or police stop secondhand can be a stressful event for young people of color, we provide evidence that the frequency of police interactions with residents in a neighborhood, as a composite of both direct and vicarious exposure to policing, is likely a dimension of the social environment that helps describe spatial variation in severe mental health outcomes among adolescents and young adults independent of area measures of socio-economic deprivation [68].

The contribution of these results to these bodies of literature must be interpreted in the context of assumptions about how crime rates, patterns of policing, and severe mental health outcomes are related, and how they are operationalized as quantitative measures. While we statistically adjusted for measures of neighborhood economic deprivation, we were unable to directly control for crime rates, as data on actual criminalized behaviors and activities are not available. Actual crime rates would be a strong confounder of the observed association. Arrest rates have been used in past research as an imperfect proxy for actual crime rates, while discretionary SQF rates are an accepted proxy for “excessive” policing. Previous analyses of NYC SQF data demonstrate that these discretionary policing encounters disproportionately involved Black and Hispanic New Yorkers, even after adjusting for prior neighborhood- and race-specific arrest rates [69]. In our study, sensitivity analyses demonstrated that the observed association (both the main effect and the interaction term) was stronger when the exposure was limited to SQF encounters and criminal summonses rather than the sum of all policing incidents. The use of this proxy for more discretionary forms of policing, which excludes encounters resulting in arrests, undermines the intrepretation that our findings are merely a function of consistent and proportional police response to serious criminalized activity across space and time. While this does not completely mitigate confounding from crime rates, it does help to isolate the role of “excessive” policing in the observed association.

Given that the outcome used in this study was total hospitalization time, the observed association may have been driven by hospital admissions involving longer periods of treatment or confinement prior to discharge. While this may indicate an association of exposure to policing with more severe exacerbations of psychiatric conditions, inpatient hospital admission and length of stay represent multiple interacting phenomena, including the incidence and severity of psychiatric morbidity, access to care, healthcare decisions made by patients and their caregivers, and the protocols and clinical decision-making of healthcare facilities and their staff. The effect modification we observed by neighborhood racial composition may be partially explained by differences in treatment norms or protocols in hospitals serving minoritized neighborhoods, or propensity of medical staff and providers to treat youth from these neighborhoods differently than their counterparts from white neighborhoods, consistent with historically disproportionate institutionalization of Black people [70]. Indeed, institutional psychiatric care has been described as inter-related to systems of incarceration, with the labeling and confinement of people with mental illness serving as a form of social control complementary to criminalization as a means to maintain social order [71, 72]. Despite the methodological and conceptual complexities involved, we encourage future research examining the geographic variation in utilization of inpatient psychiatric services among adolescents and young adults to further explore the role of exposure to policing and carceral systems as an important aspect of the social environment of urban youth.

Under the “broken windows” approach to policing, neighborhoods targeted by police to “pre-empt” criminalized activity are those with high rates of unemployment and deteriorating infrastructure, phenomena which are embedded in more long-term processes of “organized abandonment” in which disinvestment in social infrastructure is coupled with increasing investment in law enforcement and carceral infrastructure, replacing social services with surveillance and punishment over decades [42]. As evidence for the relationship between policing and physical and mental health continues to mount, disentangling direct and vicarious exposure to policing from these other entrenched social, economic, and political processes that shape neighborhoods, and establishing the directionality of observed associations, will require more explicitly causal study designs and methods, particular those that identify potential mechanisms [27, 62, 68]. Examples include a difference-in-differences analysis by Bor and colleagues, which found the self-reported mental health of Black Americans to be causally related to indirect exposure to police killings of unarmed Black people [73], and a laboratory-based study which found that both self-reported affect and autonomic nervous system function of young Black adults were affected by exposure to photos of police violence against Black people [74]. At the same time, in addition to documenting the public health consequences of exposure to policing and the carceral state, researchers can develop a more complete causal picture by studying the effects of policies and programs that move resources away from policing and toward social and economic services and investments. [75].

Several additional limitations related to the data used in this study deserve further consideration. First, while the use of administrative data for the exposure, outcome, sociodemographic covariates, and geographic boundaries demonstrates that these data sources can generate large, real-world datasets useful for population health research, the instruments and systems used to collect and organize these data were not designed for the systematic study of health-related phenomena. For example, the use of Zip Code Tabulation Areas to represent neighborhoods is not ideal as they may not represent socially meaningful neighborhood boundaries or geographic units necessarily relevant to the association under study, such as may be the case with police precincts [76]. This level of geospatial aggregation was implemented to accommodate linkage among the data sources used, which had ZCTA as the most granular common geography availabile. Second, covariates were not available at the same temporal resolution as the exposure and outcome, as they were only available as multi-year estimates. However, when the policing and hospitalization data were aggregated by year instead of month, at a more similar time resolution as the covariate data, models yielded similar results (not shown). Third, our study was limited to a 9-year period covering the implementation of New York City’s Stop, Question, and Frisk policy and the generalizability of our results may be limited to the more recent time period after the law was deemed unconstitutional. However, this enabled us to take advantage of the granular data collected on a highly discretionary form of police activity. Fourth, some policing incidents may be directly related to psychiatric emergencies; if the involvement of law enforcement in the response to mental health emergencies is substantial, this could inflate our estimates. However, we do not suspect this to be the case, since the use of all policing incidents and the inclusion of residents of all ages in our measure of total policing would most likely bias the observed association towards the null, compared with the use of a policing measure which was more responsive to mental health service calls among youth. Fifth, our use of ICD-9 coding to identify psychiatric hospitalizations may result in misclassification, as the application of an ICD code to a discharge record does not equate to a formal psychiatric diagnosis or a definitive adjudication that a mental health emergency was the underlying reason for the admission or length of stay. This concern is mitigated by the sensitivity analysis which shows stronger results when including only hosptializations with a psychiatric ICD code as the primary diagnosis on the discharge record. Furthermore, the lack of analytic differentiation between different mental health or substance use disorder codes may bias the results towards the null if hospitalization for certain disorders is more likely to be related to levels of community policing than others, or away from the null if the ICD codes include medical or behavioral issues which represent upstream drivers of increased police activity in a neighborhood rather than its downstream effects. For example, the inclusion of ICD codes for substance use could inflate the observed association to reflect that police are more likely to patrol areas where youth are more likely to consume controlled substances. However we find this unlikely to be the case due to the fact that a small share of admissions in this study (< 10%) had a primary diagnosis of substance use or alcohol use disorder. Lastly, while we drew on theory and prior empirical research to control for area-level social and economic measures relevant to the exposure and outcome under study, there may be unmeasured sources of time-varying confounding.

Our findings support what hyperpoliced communities have long known: exposure to aggressive policing is harmful to individual and community health [77, 78]. Given the rise in prevalence of adolescent psychiatric disorders over the past decade, preventing and reducing the burden of mental illness will require confronting its social and structural determinants in addition to treating individuals [79]. The World Health Organization’s commission on Social Determinants of Health has described social determinants broadly as “the fundamental global and national structures of social hierarchy and the socially determined conditions these create in which people grow, live, work, and age.” [80] In contexts of mass criminalization and incarceration, policies and practices of aggressive policing must be considered as a source of harm that exacerbates deeply entrenched, racialized social and material inequities across neighborhoods.

## Electronic supplementary material

Below is the link to the electronic supplementary material.

Supplementary Material 1
