# Supplementary material for: Maintaining disorder: estimating the association between policing and psychiatric hospitalization among youth in New York City by neighborhood racial composition, 2006–2014
Source: Soc Psychiatry Psychiatr Epidemiol. 2024 Aug 1;60(1):125–37. doi: 10.1007/s00127-024-02738-7 (PMC11790728; doi:10.1007/s00127-024-02738-7)
Supplement: Supplementary file 1 — Supplementary Material 1 [file 127_2024_2738_MOESM1_ESM.docx]

Table S-1: Most common primary diagnoses among adolescents and young adults in New York City, 2006-2014. Expressed as the proportion of total psychiatric inpatient admissions, the median days per admission, and the rate of both admissions and inpatient days per 1,000 residents aged 10-24 per year.

| ICD-9 Category of Primary Diagnosis | ICD-9 Code | % Of All Psychiatric Inpatient Admissions | Admissions  per 1,000 | Inpatient Days per 1,000 | Inpatient Days per Admission |
| --- | --- | --- | --- | --- | --- |
| Episodic mood disorders | 296 | 20.7% | 2.7 | 39.6 | 10 |
| Schizophrenic disorders | 295 | 9.7% | 1.3 | 32.8 | 17 |
| Other nonorganic psychoses | 298 | 4.7% | 0.6 | 9.6 | 11 |
| Drug dependence | 304 | 3.5% | 0.5 | 2.9 | 5 |
| Epilepsy and recurrent seizures | 345 | 3.1% | 0.4 | 1.7 | 3 |
| Drug-induced mental disorders | 292 | 2.8% | 0.4 | 2.4 | 5 |
| Depressive disorder, not elsewhere classified | 311 | 2.6% | 0.3 | 3.4 | 7 |
| Disturbance of conduct not elsewhere classified | 312 | 2.6% | 0.3 | 5.6 | 9 |
| Adjustment reaction | 309 | 2.6% | 0.3 | 2.8 | 5 |
| General Symptoms | 780 | 1.8% | 0.2 | 0.9 | 3 |

Table S-2: Estimates from fully-adjusted multilevel models demonstrating the association between policing rates and the rate of psychiatric hospitalization time, and effect modification by neighborhood racial composition. Restricted to inpatient admissions with a psychiatric ICD-9 code as primary diagnosis.

|  | **Model 1** | | **Model 2** | |
| --- | --- | --- | --- | --- |
| *Predictors* | *Incidence Rate Ratio (IRR)* | *CI* | *Incidence Rate Ratio (IRR)* | *CI* |
| Z-Score of Policing Rate | 1.04 ^**^ | 1.01 – 1.07 | 0.97 | 0.91 – 1.04 |
| Black Residents (2nd Quartile) |  |  | 1.15 ^**^ | 1.04 – 1.28 |
| Black Residents (3rd Quartile) |  |  | 1.29 ^***^ | 1.12 – 1.48 |
| Policing Rate X Black Residents (2nd Quartile) |  |  | 1.08 | 1.00 – 1.17 |
| Policing Rate X Black Residents (3rd Quartile) |  |  | 1.12 ^**^ | 1.03 – 1.21 |
| ** p<0.05   ** p<0.01   *** p<0.001* | | | | |

Table S-3: Estimates from fully-adjusted multilevel models demonstrating the association between policing rates and the rate of psychiatric hospitalization time, and effect modification by neighborhood racial composition. Adjusted for material deprivation and social fragmentation indices.

|  | **Model 1** | | **Model 2** | |
| --- | --- | --- | --- | --- |
| *Predictors* | *Incidence Rate Ratio (IRR)* | *CI* | *Incidence Rate Ratio (IRR)* | *CI* |
| Z-Score of Policing Rate | 1.03 ^**^ | 1.01 – 1.06 | 0.96 | 0.89 – 1.02 |
| Black Residents (2nd Quartile) |  |  | 1.14 ^*^ | 1.03 – 1.27 |
| Black Residents (3rd Quartile) |  |  | 1.30 ^***^ | 1.13 – 1.49 |
| Policing Rate X Black Residents (2nd Quartile) |  |  | 1.08 | 1.00 – 1.17 |
| Policing Rate X Black Residents (3rd Quartile) |  |  | 1.13 ^**^ | 1.04 – 1.22 |
| ** p<0.05   ** p<0.01   *** p<0.001* | | | | |

Table S-4: Estimates from fully-adjusted multilevel models demonstrating the association between policing rates and the rate of psychiatric hospitalization time, and effect modification by neighborhood racial composition. Measure of policing restricted to stop, question, and frisk encounters and criminal summonses

|  | **Model 1** | | **Model 2** | |
| --- | --- | --- | --- | --- |
| *Predictors* | *Incidence Rate Ratio (IRR)* | *CI* | *Incidence Rate Ratio (IRR)* | *CI* |
| Z-Score of Policing Rate | 1.02 ^*^ | 1.00 – 1.04 | 0.95 ^*^ | 0.90 – 1.00 |
| Black Residents (2nd Quartile) |  |  | 1.16 ^**^ | 1.05 – 1.29 |
| Black Residents (3rd Quartile) |  |  | 1.31 ^***^ | 1.14 – 1.50 |
| Policing Rate X Black Residents (2nd Quartile) |  |  | 1.10 ^**^ | 1.03 – 1.17 |
| Policing Rate X Black Residents (3rd Quartile) |  |  | 1.11 ^**^ | 1.04 – 1.18 |
| ** p<0.05   ** p<0.01   *** p<0.001* | | | | |

Table S-5: Estimates from fully-adjusted multilevel models demonstrating the association between policing rates and counts of psychiatric hospitalizations, and effect modification by neighborhood racial composition.

|  | **Model 1** | | **Model 2** | |
| --- | --- | --- | --- | --- |
| *Predictors* | *Incidence Rate Ratio (IRR)* | *CI* | *Incidence Rate Ratio (IRR)* | *CI* |
| Z-Score of Policing Rate | 1.03 ^***^ | 1.02 – 1.05 | 1.02 | 0.97 – 1.06 |
| Black Residents (2nd Quartile) |  |  | 1.13 ^***^ | 1.06 – 1.21 |
| Black Residents (3rd Quartile) |  |  | 1.19 ^***^ | 1.08 – 1.31 |
| Policing Rate X Black Residents (2nd Quartile) |  |  | 1.02 | 0.96 – 1.07 |
| Policing Rate X Black Residents (3rd Quartile) |  |  | 1.02 | 0.97 – 1.07 |
| ** p<0.05   ** p<0.01   *** p<0.001* | | | | |

Table S - 6: Estimates from fully-adjusted multilevel models demonstrating the association between policing rates and the rate of psychiatric hospitalization time, and effect modification by neighborhood racial composition. Restricted to inpatient hospitalizations < 30 days

|  | **Model 1** | | **Model 2** | |
| --- | --- | --- | --- | --- |
| *Predictors* | *Incidence Rate Ratio (IRR)* | *CI* | *Incidence Rate Ratio (IRR)* | *CI* |
| Z-Score of Policing Rate | 1.04 ^***^ | 1.01 – 1.06 | 1.01 | 0.96 – 1.05 |
| Black Residents (2nd Quartile) |  |  | 1.14 ^**^ | 1.05 – 1.24 |
| Black Residents (3rd Quartile) |  |  | 1.28 ^***^ | 1.14 – 1.43 |
| Policing Rate X Black Residents (2nd Quartile) |  |  | 1.02 | 0.97 – 1.08 |
| Policing Rate X Black Residents (3rd Quartile) |  |  | 1.04 | 0.98 – 1.10 |
| ** p<0.05   ** p<0.01   *** p<0.001* | | | | |

Table S – 7a: Estimates from fully-adjusted multilevel models demonstrating the association between policing rates and the rate of psychiatric hospitalization time, and effect modification by neighborhood racial composition. In these models, the exposure predicts future hospitalizations with a 1 month lag.

|  | **Model 1** | | **Model 2** | |
| --- | --- | --- | --- | --- |
| *Predictors* | *Incidence Rate Ratio (IRR)* | *CI* | *Incidence Rate Ratio (IRR)* | *CI* |
| Policing Rate | 1.003 ^**^ | 1.001 – 1.004 | 1.001 | 0.998 – 1.004 |
| Black Residents (2nd Quartile) |  |  | 1.014 | 0.903 – 1.138 |
| Black Residents (3rd Quartile) |  |  | 0.815 ^*^ | 0.684 – 0.971 |
| Policing Rate X Black Residents (2nd Quartile) |  |  | 1.001 | 0.997 – 1.006 |
| Policing Rate X Black Residents (3rd Quartile) |  |  | 1.003 | 0.998 – 1.007 |
| ** p<0.05   ** p<0.01   *** p<0.001* | | | | |

Table S – 7b: Estimates from fully-adjusted multilevel models demonstrating the association between policing rates and the rate of psychiatric hospitalization time, and effect modification by neighborhood racial composition. In these models, the exposure predicts future hospitalizations with a 3 month lag.

|  | **Model 1** | | **Model 2** | |
| --- | --- | --- | --- | --- |
| *Predictors* | *Incidence Rate Ratio (IRR)* | *CI* | *Incidence Rate Ratio (IRR)* | *CI* |
| Policing Rate | 1.003 ^**^ | 1.001 – 1.005 | 1.002 | 0.999 – 1.005 |
| Black Residents (2nd Quartile) |  |  | 1.003 | 0.889 – 1.131 |
| Black Residents (3rd Quartile) |  |  | 0.847 | 0.709 – 1.013 |
| Policing Rate X Black Residents (2nd Quartile) |  |  | 0.999 | 0.995 – 1.004 |
| Policing Rate X Black Residents (3rd Quartile) |  |  | 1.003 | 0.999 – 1.007 |
| ** p<0.05   ** p<0.01   *** p<0.001* | | | | |

Table S - 8: Estimates from fully-adjusted multilevel models demonstrating the association between policing rates and the rate of psychiatric hospitalization time, and effect modification by neighborhood racial composition. In these models, the exposure predicts future hospitalizations with a 1 month lag.

|  | **Model 1** | | **Model 2** | |
| --- | --- | --- | --- | --- |
| *Predictors* | *Incidence Rate Ratio (IRR)* | *CI* | *Incidence Rate Ratio (IRR)* | *CI* |
| Policing Rate | 1.002 ^*^ | 1.000 – 1.004 | 0.999 | 0.995 – 1.003 |
| Black Residents (2nd Quartile) |  |  | 1.078 | 0.980 – 1.186 |
| Black Residents (3rd Quartile) |  |  | 1.163 ^*^ | 1.025 – 1.320 |
| Policing Rate X Black Residents (2nd Quartile) |  |  | 1.002 | 0.998 – 1.007 |
| Policing Rate X Black Residents (3rd Quartile) |  |  | 1.005 ^*^ | 1.001 – 1.010 |
| ** p<0.05   ** p<0.01   *** p<0.001* | | | | |
